# Functional innovations of three chronological mesohexaploid *Brassica rapa* genomes

**DOI:** 10.1186/1471-2164-15-606

**Published:** 2014-07-18

**Authors:** Jungeun Kim, Jeongyeo Lee, Jae-Pil Choi, Inkyu Park, Kyungbong Yang, Min Keun Kim, Young Han Lee, Ill-Sup Nou, Dae-Soo Kim, Sung Ran Min, Sang Un Park, HyeRan Kim

**Affiliations:** Korea Research Institute of Bioscience and Biotechnology (KRIBB), 125 Gwahangno, Yuseong-gu, Daejeon, 305-806 Republic of Korea; Biosystems & Bioengineering, University of Science and Technology, 217 Gajeong-ro, Yuseong-gu, Daejeon, Republic of Korea; College of Agriculture and Life Science, Chungnam National University, 99 Daehak-ro, Yuseong-gu, Daejeon, 305-764 Republic of Korea; Division of Environment-Friendly Research, Gyeongsangnam-do Agricultural Research and Extension Services, Jinju, 660-360 Republic of Korea; Department of Horticulture, Sunchon National University, Suncheon, 540-742 Republic of Korea

**Keywords:** *Brassica rapa*, Chronological genomes, Fast-evolving genes, Single-retention genes, Multi-retention genes

## Abstract

**Background:**

The Brassicaceae family is an exemplary model for studying plant polyploidy. The Brassicaceae knowledge-base includes the well-annotated *Arabidopsis thaliana* reference sequence; well-established evidence for three rounds of whole genome duplication (WGD); and the conservation of genomic structure, with 24 conserved genomic blocks (GBs). The recently released *Brassica rapa* draft genome provides an ideal opportunity to update our knowledge of the conserved genomic structures in *Brassica*, and to study evolutionary innovations of the mesohexaploid plant, *B. rapa*.

**Results:**

Three chronological *B. rapa* genomes (recent, young, and old) were reconstructed with sequence divergences, revealing a trace of recursive WGD events. A total of 636 fast evolving genes were unevenly distributed throughout the recent and young genomes. The representative Gene Ontology (GO) terms for these genes were ‘stress response’ and ‘development’ both through a change in protein modification or signaling, rather than by enhancing signal recognition. In retention patterns analysis, 98% of *B. rapa* genes were retained as collinear gene pairs; 77% of those were singly-retained in recent or young genomes resulting from death of the ancestral copies, while others were multi-retained as long retention genes. GO enrichments indicated that single retention genes mainly function in the interpretation of genetic information, whereas, multi-retention genes were biased toward signal response, especially regarding development and defense. In the recent genome, 13,302, 5,790, and 20 gene pairs were multi-retained following *Brassica* whole genome triplication (WGT) events with 2, 3, and 4 homoeologous copies, respectively. Enriched GO-slim terms from *B. rapa* homomoelogues imply that a major effect of the *B. rapa* WGT may have been to acquire environmental adaptability or to change the course of development. These homoeologues seem to more frequently undergo subfunctionalization with spatial expression patterns compared with other possible events including nonfunctionalization and neofunctionalization.

**Conclusion:**

We refined Brassicaceae GB information using the latest genomic resources, and distinguished three chronologically ordered *B. rapa* genomes. *B. rapa* genes were categorized into fast evolving, single- and multi-retention genes, and long retention genes by their substitution rates and retention patterns. Representative functions of the categorized genes were elucidated, providing better understanding of *B. rapa* evolution and the *Brassica* genus.

**Electronic supplementary material:**

The online version of this article (doi:10.1186/1471-2164-15-606) contains supplementary material, which is available to authorized users.

## Background

The genus *Brassica* belongs to the Brassiceae tribe, Brassicaceae family, Brassicales order. The genus contains 38 species and several varieties, as well as numerous hybrids. The six major *Brassica* species are described by the “Triangle of U”: three diploid genomes of *B. rapa* (AA genome, 2n = 2x = 20), *B. nigra* (BB genome, 2n = 2x = 16), and *B. oleracea* (CC genome, 2n = 2x = 18), formed into the three amphidiploid plants *B. juncea* (AABB genome, 2n = 4x = 36), *B. napus* (AACC genome, 2n = 4x = 38), and *B. carinata* (BBCC genome, 2n = 4x = 34) through interspecific hybridization [1]. Genomic orders are conserved between diploid and amphidiploid *Brassica* species according to marker-based studies [2–4]. Therefore, the construction of reference *Brassica* A, B, and C genomes provides a framework for many various *Brassica* species. The whole genome sequence (WGS) of *Brassica* A has been released with the *B. rapa* ssp. *pekinensis* line Chiifu-401-42 [5] and the WGS of *B. oleraceae* (C genome) will be available in the near future [6]. These valuable resources enable us to elucidate species identity as a consequence of whole genome triplication (WGT), to discover molecular markers useful in breeding, and to profile gene variants, all further enhancing our understanding of evolution within the group.

One of the more interesting outcomes of the increase in plant genomic research is the plethora of species expansion and diversification studies available. The polyploidy event, known as whole genome duplication (WGD), is a major contributor to genome evolution and species radiation through its ability to increase the odds of obtaining new functions in a genome [7–9]. The Brassicaceae (formerly Cruciferae) family is an exemplary model for studying polyploidy events because the well annotated *Arabidopsis thaliana* (*A. thaliana*) genome exists as a reference [10], with its well supported three rounds of WGD (*At-*α, *At-*β, and *At-*γ) [11]. In addition, sub-classification of the Brassicaceae species is relatively clear for lineages I–III [12]. The genus *Brassica* experienced an additional WGT around 13–17 million years ago (Mya) [13, 14]. The timing of this WGT makes *Brassica* an important model genus for evolutionary study because genomic collinearity among the species is maintained with their ancestral genome, a decisive factor in estimating ancestral genomes. The model plants, *A. thaliana* and *B. rapa*, belong to the core-Brassicaceae lineage I and II, respectively [12]. Conservation of genomic structure from the Ancestral Crucifer Karyotype (ACK; n = 8) has been reported in Brassicaceae [15], and 24 conserved genomic blocks (GBs) based on *A. thaliana* loci have also been established [16]. The common ancestor of lineage II in Brassicaceae (Proto-Calepineae Karyotype (PCK); n = 7) experienced chromosomal reduction [17]. Additional translocation was also experienced translocation-PCK (tPCK) in several genera of the Brassicaceae lineage II, including the genus *Brassica* [18]. Information about conserved GBs and their loci makes it easy to compare genomic structures as well as gene expansions related to *Brassica* diversity.

After WGD, a plant genome is reorganized via chromosomal rearrangements, excessive gene fractionation, and epigenetic changes [9, 19]. In *Arabidopsis*, 80 Mb and 33.2 Mb of the genome originated from recent (α-WGD), and from old (βγ-WGD) polyploidy events, respectively, according to a recent synonymous genomic blocks substitution analysis [20]. After reorganization resulting from WGD, genomes preferentially retain genes or gene families [21–23]. In *Arabidopsis* these genes have been reported to be dosage sensitive and to be functionally involved in transcriptional and/or developmental regulation [24], biological networks and signal cascades [22, 24], as well as in protein complexes [23]. Furthermore, longer retained genes contribute to species radiations by subfunctionalization or neofunctionalization after polyploidy [25]. In the *B. rapa* genome multi-retained genes have been reported to be involved in environmental stress, hormone response, transcription factors (TFs), ribosome structure, cell wall, and cytoskeleton organization [5]. Specifically, auxin-related gene families, which control a plant’s growth and morphological development, are over-retained in the *B. rapa* genome, which is an indicator that these genes are potential contributors to morphological diversification [5]. Multi-retained genes possessing biased function are not specific to *B. rapa*, but are also common in other duplicated genomes [24]. The innovative features of the *B. rapa* genome introduced by its recent WGT, and the major fate of those duplicated genes in the genome are not yet fully understood.

In this study we aim to refine GB information using the latest genomic data, and to distinguish the historic *B. rapa* genomes chronologically for further studies. Fast evolving and multi-retention genes have been elucidated, and genome innovations after the WGT event are discussed. This analysis will contribute to understanding *B. rapa* evolution in general, as well as suggest future experimental designs for studying *Brassica* diversity.

## Results

### Reconstruction of three chronological *B. rapa*genomes with 24 refined genomic blocks

**Figure 1:**
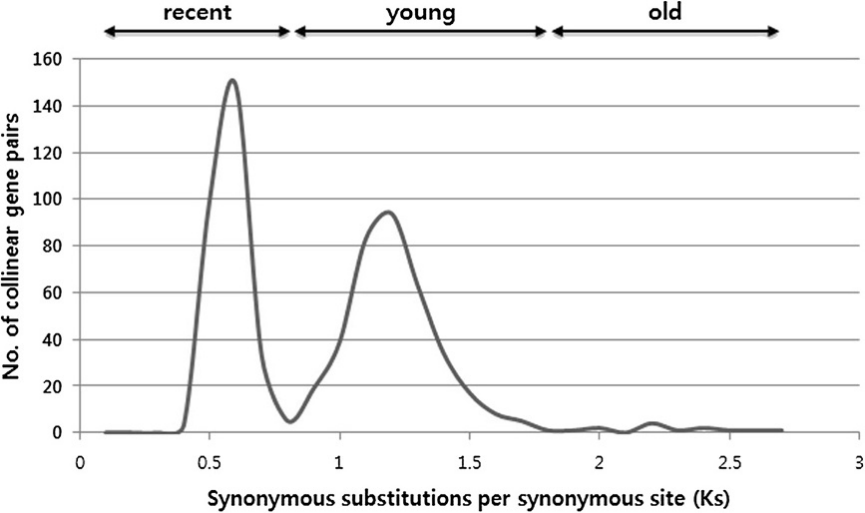
**The distribution of the average** *K*_*s*_ **values of the collinear gene pairs between** *A. thaliana* **and** *B. rapa* **genome.**

**Figure 2:**
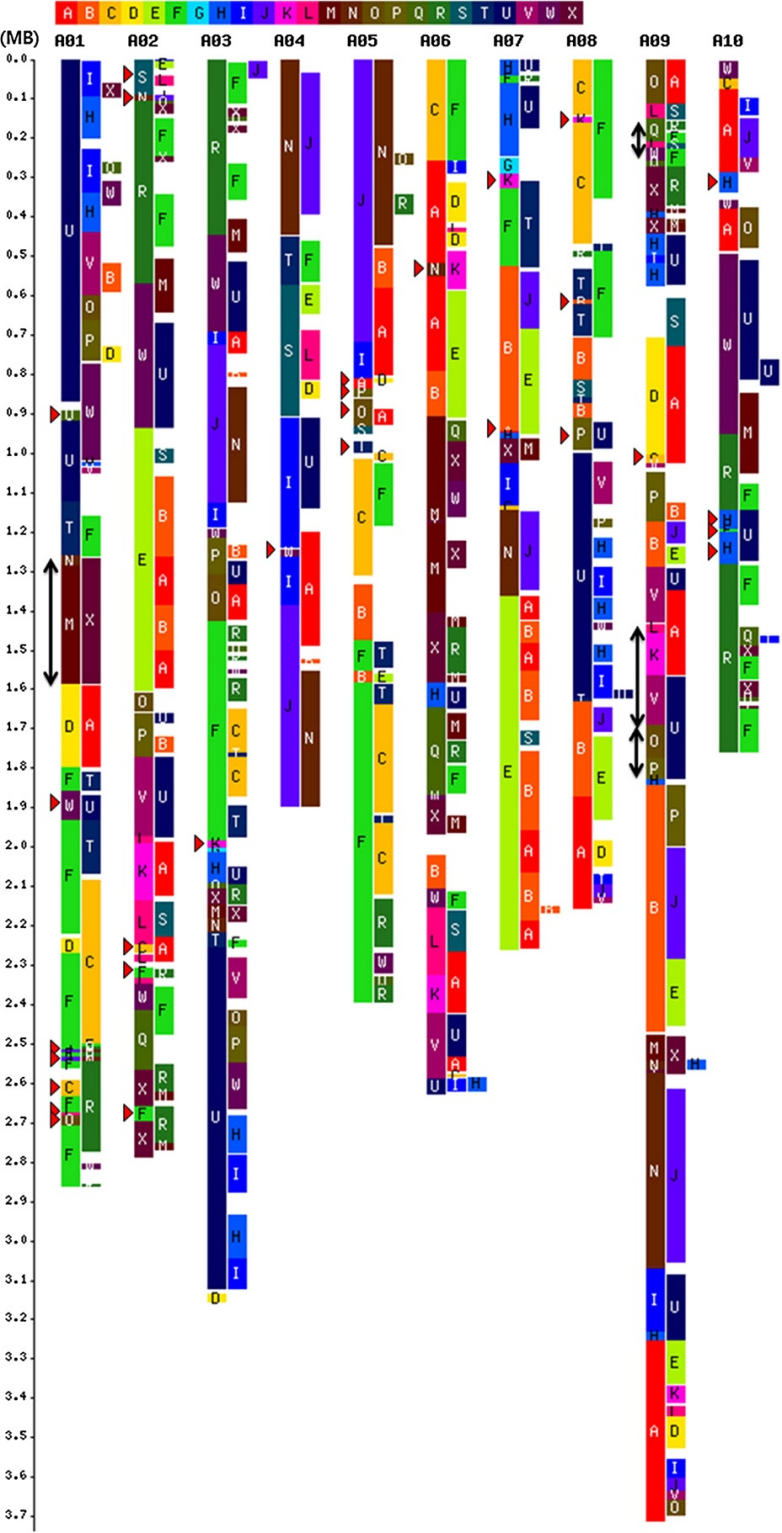
**Three reconstructed periodical** *B. rapa* **genomes based on refined 24 GBs.** The left bar represents 0.1 Mb scale of the *B. rapa* genome and A-X rectangles in the upper part represent 24 genomic blocks (GB). Each chromosome represents three periodical genomic segments distinguished by average synonymous substitution rates of genomic segments (from left, recent, young, and old). The red triangles (►) and arrows (↕) in the left side of the GB map represent newly identified GBs in this study and an inverted order of GBs compared to Cheng et al. (2013) [18], respectively. The structural difference between Cheng et al. (2013) [18] and our map may be arisen by using different version of the *B. rapa* genome.

We identified putative homologous chromosomal segments between *A. thaliana* and *B. rapa* genomes using the MCScan algorithm. A total of 683 syntenic segments were identified between the two genomes with 36,683 collinear gene pairs. Sequence divergence between the collinear gene pairs, calculated as synonymous substitutions per synonymous site (*K*_*s*_), ranged from 0.1 to 6.2, with an average value of 0.51 (Additional file 1). The average *K*_*s*_ values of the collinear gene pairs in each syntenic segment were distributed into three waves, which were attributed to traces of paleo-WGD and recent triplication events (Figure 1). The first wave contained 302 syntenic segments with 29,239 collinear gene pairs; we named these “recent” segments (Table 1). The second and third waves corresponded to “young” and “old” segments; and contained 366 syntenic segments, with 7,335 collinear gene pairs; and 15 syntenic segments, with 109 collinear gene pairs; respectively. Approximately 5–13 times more collinear genes were identified in recent syntenic segments than in other older segments. Twenty-four Brassicaceae GBs in the *Arabidopsis* genome were refined by identifying collinear genes between *A. thaliana* and *B. rapa* with evidence of the syntenic segment’s continuity. This represented an expansion to the GBs proposed by Schranz et al. [16] and Cheng et al. [18]. A total of 24 GBs excluding “G”, “R”, and “W” blocks were expanded by 0.01–2.52 Mb, assigning 2,997 more genes for a total of 113.34 Mb (99.41%) of the GBs defined in the *Arabidopsis* genome (Table 2). Finally, three historic *B. rapa* genomes, identified by the sequence divergence values of their syntenic segments, were reconstructed, clearly showing the trace of recursive WGD events (Figure 2).

**Table 1:** **Classification of three chronological genomes by sequence divergence of the collinear gene pairs** *

	Recent	Young	Old
Avg.*K*_*s*_	0.53	1.17	2.19
**Syntenic segment information**			
No. of syntenic segments	302	366	15
No. of collinear protein pairs	29,239	7,335	109
**Information of** *B. rapa* **genome segment**			
Size of genome segment	250,327,645	203,058,429	5,49,677
(%)	(97.69)	(79.24)	(2.14)
No. of integrated GBs	172	223	14
Average collinear gene pairs	1,217	318	5
No. of distinct *A. thaliana* genes	18,701	5,209	109
in synteny	(53.48%)	(14.90%)	(0.31%)
Collinear-pairs/Mb	107	37	10

*Detailed information about this table is represented in Additional file 2.

**Table 2:** **Reconstructed genomic blocks based on the synteny between** *A. thaliana* **and** *B. rapa*

AK	GB	Current GB*				Reconstructed GB			
		Interval (Mb)		No of genes (%)		Intervals (Mb)		No of genes (%)	
1	A	At1g01560-At1g19330	(6.48)	2,528	(7.19)	At1g01010-At1g19840	(6.87)	2,690	(7.65)
1	B	At1g19850-At1g36240	(6.73)	1,949	(5.54)	At1g19850-At1g37130	(7.27)	1,986	(5.65)
1	C	At1g43600-At1g56120	(4.84)	1,404	(3.99)	At1g43020-At1g56190	(4.88)	1,418	(4.03)
2	D	At1g63770-At1g56530	(2.49)	744	(2.12)	At1g64670-At1g56210	(3.00)	913	(2.60)
2	E	At1g65040-At1g80420	(6.08)	2,086	(5.93)	At1g64960-At1g80950	(6.29)	2,163	(6.15)
3	F	At3g01040-At3g25520	(9.26)	3,423	(9.73)	At3g01015-At3g25520	(9.27)	3,427	(9.74)
3	G	At2g05170-At2g07690	(1.66)	229	(0.65)	At2g05170-At2g07690	(1.66)	229	(0.65)
3	H	At2g15670-At2g20900	(2.16)	669	(1.90)	At2g10940-At2g20900	(4.68)	940	(2.67)
4	I	At2g20920-At2g28910	(3.42)	1,091	(3.10)	At2g20920-At2g31035	(4.21)	1,385	(3.94)
4	J	At2g31040-At2g47730	(6.35)	2,400	(6.82)	At2g31040-At2g48150	(6.48)	2,465	(7.01)
5	K	At2g01250-At2g03750	(1.02)	326	(0.93)	At2g01060-At2g05160	(1.79)	508	(1.44)
5	L	At3g25855-At3g29770	(2.19)	531	(1.51)	At3g25540-At3g32960	(4.23)	684	(1.94)
5	M	At3g43740-At3g49970	(2.88)	789	(2.24)	At3g42180-At3g50940	(4.61)	1,001	(2.85)
5	N	At3g50950-At3g62790	(4.31)	1,678	(4.77)	At3g50950-At3g63530	(4.52)	1,771	(5.03)
6	O	At4g00030-At4g04955	(2.51)	633	(1.80)	At4g00026-At4g05450	(2.75)	708	(2.01)
6	P	At4g12070-At4g08690	(1.72)	434	(1.23)	At4g07390-At4g12620	(3.27)	595	(1.69)
6	Q	At5g28885-At5g23010	(3.20)	741	(2.11)	At5g30510-At5g23010	(3.92)	771	(2.19)
6	R	At5g23000-At5g01010	(7.70)	2,825	(8.03)	At5g23000-At5g01010	(7.70)	2,825	(8.03)
7	S	At5g41900-At5g33210	(4.31)	853	(2.42)	At5g42110- At5g32470	(4.75)	886	(2.52)
7	T	At4g12750-At4g16143	(1.64)	526	(1.50)	At4g12700-At4g16240	(1.71)	556	(1.58)
7	U	At4g16250-At4g38770	(8.90)	3,252	(9.24)	At4g16250-At4g40100	(9.39)	3,446	(9.80)
8	V	At5g42130-At5g42810	(0.33)	97	(0.28)	At5g42130-At5g47810	(2.52)	805	(2.29)
8	W	At5g47820-At5g60800	(5.10)	1,805	(5.13)	At5g47820-At5g60800	(5.10)	1,805	(5.13)
8	X	At5g60805-At5g67385	(2.42)	957	(2.72)	At5g60805-At5g67640	(2.51)	991	(2.82)
Total			(97.69)	31,971	(90.89)		(113.34)	34,968	(99.41)

*Genomic blocks updated by Cheng et al. (2013) [18]. AK: ancestral karyotype defined by Lysak et al. (2006) [15], GB: genomic block.

A total of 172 GBs were assigned to the recent genome, covering 250 Mb (98.69%) of the *B. rapa* genome by revealing 0.29 (“G” block) – 2.84 (“T” block) times of *A. thaliana* GB size (Additional file 2). There were 1,217 collinear gene pairs, preserving 53.48% of the *Arabidopsis* genome with collinearity (Table 1). The most conserved GB in terms of number of genes with collinearity was “R”, with 61.52% of the *A. thaliana* genes preserved in synteny, covering the *A. thaliana* “R” block 2.06 times. The *B. rapa* “G” block was the most fractionated, with 13.97% of the remaining collinear genes. The young genome was constructed with 203 Mb (79.24%) of *B. rapa* coverage, retaining 14.90% of *A. thaliana* collinear genes (Table 1). The most conserved and fractionated GBs in the young blocks were “A” and “Q” with 19.44% and 9.21% of *A. thaliana* genes, respectively (Additional file 2). The genomic block “G” was not detected in the young blocks. The old genome barely remained with 5.50 Mb of reconstructed blocks and 109 collinear gene pairs. The GB conservations were less intact in the older genome than in the younger genome, with 107, 37, and 10 collinear gene pairs per Mb in the recent, young, and old genomes, respectively (Table 1). Comparative analysis of GB arrangements in recent and young genomes showed that the “A”, “U”, and “F” GBs of the recent genome conservatively contained eight “O-V-J-I-C-D-L-K-E” blocks, eight “V-O-P-W-H-I-H-I” blocks, and seven “R-Q-R-W-R-C-T” block arrangements from the young genome, respectively (Figure 2). Three GBs (“H”, “K”, and “T”) in the recent genome consisted of only one GB (“U”, “A”, or “F”) from the young genome. The ancestral copies of the “G” block in the recent genome had been lost.

### Fast evolving genes in *B. rapa*genome with recursive WGD

**Figure 3:**
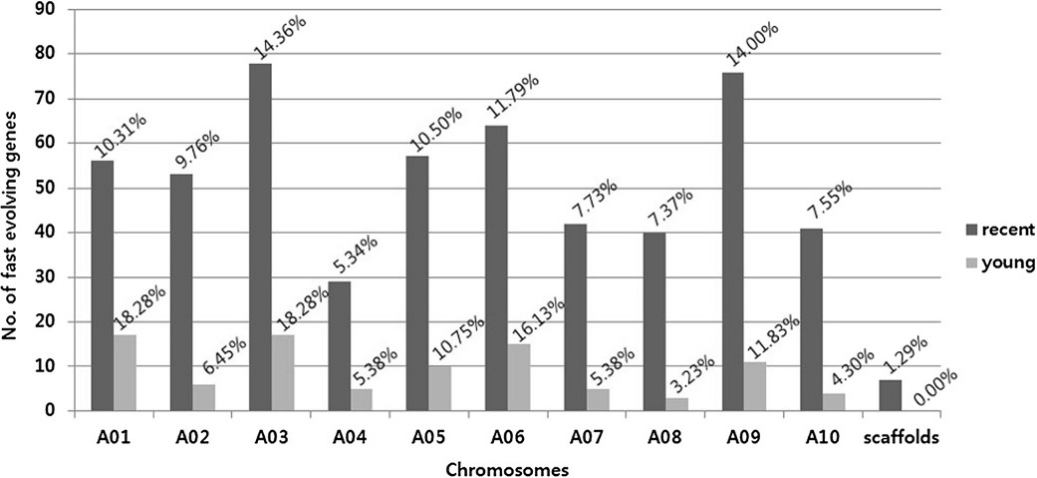
**Distribution of fast evolving genes in chromosomes.** The graphs represent the number of fast-evolving genes in recent and young genomes distributed in the *B. rapa* chromosomes and the frequencies of the genes in the chromosome are represented in each bar.

**Figure 4:**
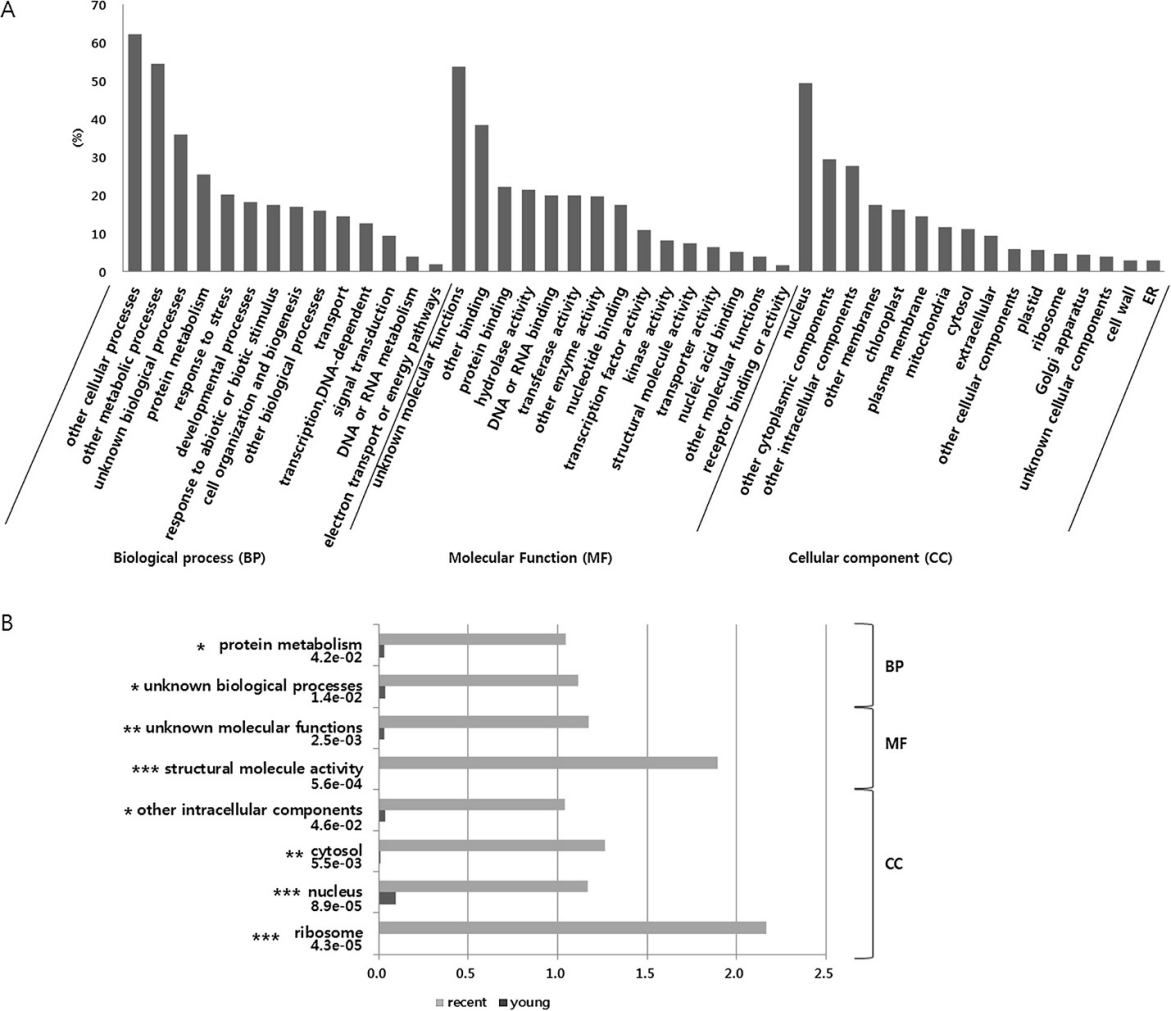
**GO slim analysis of fast evolving genes. A**. Frequency of GO-slim terms. The x-axis represents GO-slim terms classified into the biological process (BP), cellular component (CC) and molecular function (MF) while the y-axis represents % of fast evolving genes assigned to specific GO-slim categories. **B**. GO-slim enrichment analysed by fisher’s exact test and p-values estimating fisher’s exact test under the description. The x-axis represents fold ratio between the frequency of fast evolving gene in recent or young genome categorized in certain GO-slim terms and background frequency (total *B. rapa* gene are not detected in fast evolving genes) in that term. The y-axis represents GO-slim terms. Different levels of p-value (< 0.05, < 0.01, < 0.001) are represented by *, **, ***, respectively.

We selected fast evolving genes in the syntenic segments based on nucleotide substitution rates. A total of 636 fast evolving genes were identified from 265 syntenic segments by selecting genes with *K*_*s*_ values significantly higher than the average *K*_*s*_ value of their syntenic segments (p < 0.001). Among them 543 (85.38%) and 93 (14.62%) fast evolving genes were detected, from 181 recent and 84 young syntenic segments, respectively, whereas no fast evolving genes were identified in old syntenic segments (Additional file 3). The fast evolving genes were unevenly distributed throughout the *B. rapa* genome (Figure 3). In the “recent” genome, A03 chromosome had the highest number of fast evolving genes with 78 genes, while A04 had the lowest with 29 genes, not including scaffolds. In “young” genomes, A01 and A08 were the chromosomes with the most (17 genes) and the least (3 genes) fast evolving genes, respectively. The quantity of fast evolving genes in the 24 GBs were varied, with 1 (0.18% in “G”) – 67 (12.34% in “F”) genes/GB in recent blocks, and 0–11 (11.83% in “A”) genes/GB in young blocks (Additional file 3). No fast evolving genes were detected in the “G”, “L”, and “Q” GBs in the young genome. Only five genes were commonly identified as fast evolving genes in both the recent and young genomes. Fast evolving gene function was estimated using Gene Ontology (GO) annotation. A total of 631 fast evolving genes were assigned to GO terms in all hierarchies; 555 biological processes (BP), 244 molecular functions (MF) and 103 cellular components (CC) (Additional file 3). To simplify the presentation, GO terms were re-categorized into GO-slim terms. High proportions of fast evolving genes remained unknown (Figure 4A). Nevertheless, four BP-terms show moderate frequencies (>15%), ‘protein metabolism’ , ‘response to abiotic or biotic stimulus’ , ‘developmental process’ , and ‘cell organization and biogenesis.’ These genes function in ‘binding (to protein, DNA, RNA, nucleotide)’ , or as ‘enzymes (hydrolase, transferase, kinase)’ , and a small number of them are involved as ‘transporters’ or ‘receptors’ in MF-terms. In CC terms, fast evolving genes mainly localized at the ‘nucleus’ , ‘cytoplasmic’ , and ‘intracellular regions.’ To understand the representative functions of the fast evolving genes, an enrichment test was performed based on GO-slim. Despite many functions that remained unclear, several notable functions were identified such as ‘protein metabolism’ and ‘structural molecule activity’ (Figure 4B). The BP term ‘protein metabolism’ included ‘protein folding’ , ‘translation’ , ‘post-translational protein modification (myristoylation, phosphorylation, methylation, glycosylation, ubiquitination, dephosphorylation, deubiquitination, autophosphorylation)’ , ‘proteolysis’ , and ‘positive and negative regulation of serine/threonine kinase activity’. The ‘structural molecule activity’ MF terms contains ‘constituent of ribosome.’ Despite high frequencies of ‘stress’ and ‘biotic/abiotic response’ genes, terms such as ‘receptor binding/activity’ and ‘transporter’ , which facilitate the recognition and transportation of environmental signals, were observed at relatively lower frequencies in MF terms (Figure 4A). The term ‘plasma membrane’ , which indicates a recognition function, was not represented (Figure 4B).

### Functional bias according to gene retention patterns after recursive WGD

We investigated the retention patterns of the 36,638 collinear gene pairs for the GO term ‘gene birth and death’ in the three chronological genomes (Table 3). The results show that 29,784 (72.61%) of *B. rapa* genes had more than one collinear gene pair with *A. thaliana*; 11,235 (27.39%) of them had lost collinearity. The collinear gene pairs were classified into eight groups based on their retention patterns, as shown in Table 3. Most collinear gene pairs were observed in the recent genome, and 76.58% of them (22,371) were retained in the recent genome following the death of their ancestral copy (Index 1). The remaining 6,841 gene pairs retained older copies of young and/or old genomes (Indexes 2–4), and are defined as long retention genes. The higher standard deviation of *K*_*s*_ (0.98) in Index 2 compared to Index 1 (0.31) demonstrated a longer retention period for the gene pairs. However, the standard deviation of *K*_*s*_ values for other multi-retention gene pairs in Indexes 3–4 showed no significant difference with those of the single retention gene pairs because of the small number of gene pairs analyzed. The retention rate of *A. thaliana* paralogues between young (6,740, 98.77%) and old (84, 1.23%) genomes was similar to the retention rate of *B. rapa* genes in recent (29,212, 98.08%) and old (572, 1.92%) genomes. This suggests that approximately 98% synteny is generated from the direct ancestor’s gene copies, while 1–2% is generated from older ancestor gene copies.

**Table 3:** **Classification of divergence time of** *B. rapa* **gene based on** *A. thaliana* **collinear pairs**

Index	Retention patterns ^a^			Standard deviation of*K*_*s*_values			No. of *B. rapa*proteins (%)
	Recent	Young	Old	Recent	Young	Old	
1	O	X	X	0.31	-	-	22,371 (54.54)
2	O	O	X	0.98	1.31	-	6,740 (16.43)
3	O	X	O	0.29	-	0.86	84 (0.20)
4	O	O	O	0.28	0.81	0.83	17 (0.04)
5	X	X	X	-	-	-	11,235 (27.39)
6	X	O	X	-	0.62	-	564 (1.37)
7	X	X	O	-	-	0.85	4 (0.01)
8	X	O	O	-	0.78	0.30	4 (0.01)

^a^Presence (O) and/or absence (X) of collinear gene pairs in recent, young and old genomic segments. ‘-‘ denotes absence of collinear gene pairs.

**Figure 5:**
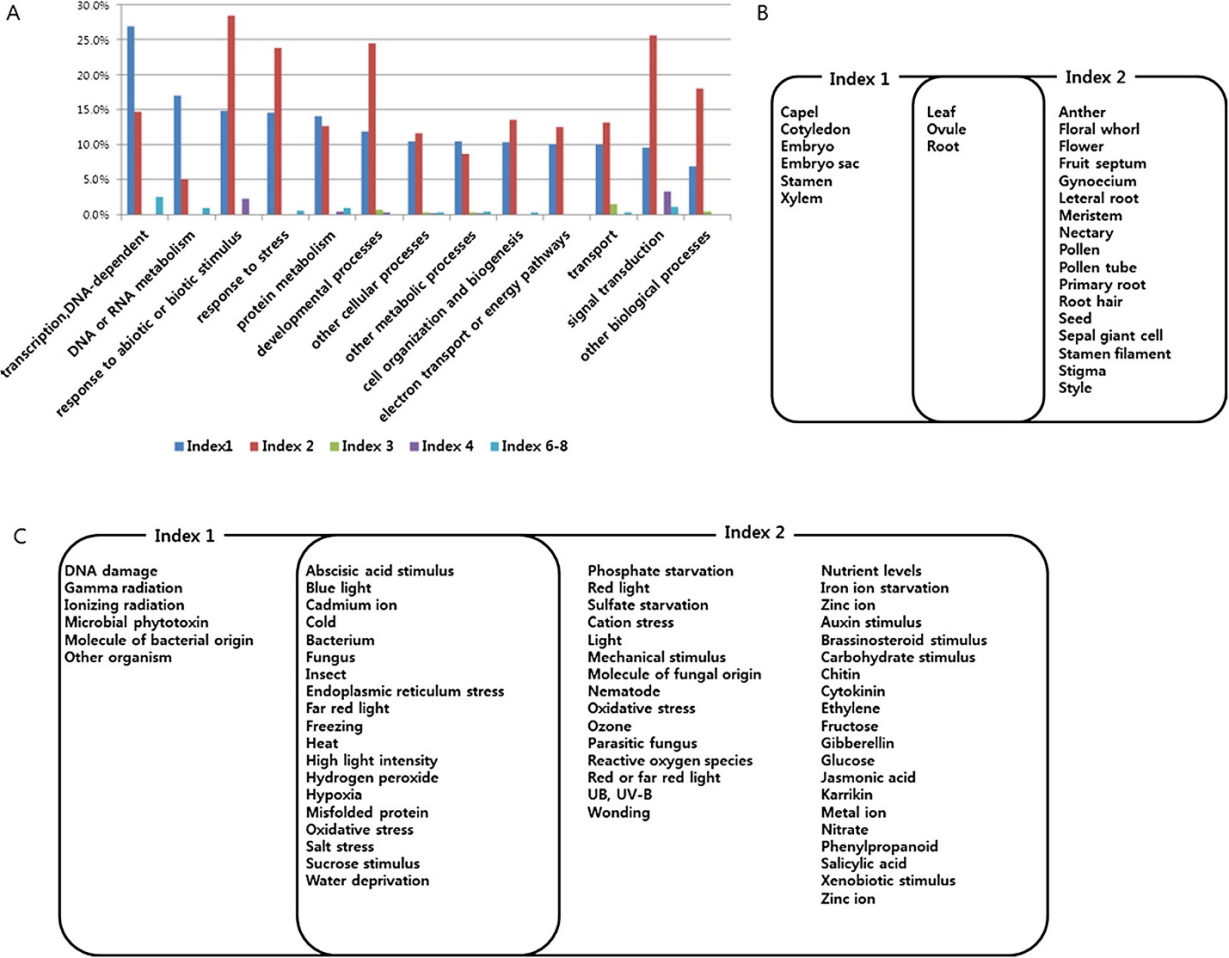
**Functional bias according to gene birth and death patterns. A**. Frequency of enriched GO terms re-categorized into the GO-slim terms. X-axis represents GO-slim description and y-axis represents percentage of enriched GO-slim terms divided by total number of GO-slim terms in specific GO-slim categories. **B**. Organ distributions of development processes enriched in index 1 and/or index 2. **C**. Stress or biological process related genes which are specifically enriched in Index 1 and/or Index 2.

Functional bias depending on gene retention patterns was investigated using GO term analysis. We detected 304, 408, 10, 14, and 19 GO terms enriched in Indexes 1, 2, 3, 4, and 6, respectively (Additional file 4). However, there were no enriched GO terms detected in Index 7 and 8 due to their small numbers of gene pair datasets. The enriched GO terms were re-categorized into GO-slim terms (Figure 5A). The major frequent GO terms in Index 1 (recent specific genes) were ‘DNA-dependent transcription’ , ‘DNA or RNA metabolism’ , ‘protein metabolism’ , and ‘other metabolic process;’ while those in Index 2 (sharing recent and young genomes as multi-retention genes) were ‘stress or biotic/abiotic response genes’ , ‘developmental process’ , ‘cellular/biological process’ , ‘cell organization and biogenesis’ , ‘transport’ , and ‘signal transduction.’ Despite a low occurrence of GO terms in Index 3 (frequent in ‘transport’ and ‘development process’) and Index 4 (frequent in ‘biotic/abiotic stimulus’ and ‘signal transduction’), GO enrichments in these gene pairs showed patterns similar to Index 2. However, the enrichment pattern observed with gene pairs in Index 6 (young specific genes) was similar to that of Index 1 (Additional file 4). This result suggests that genes retained in the recent or young genomes as single retention genes mainly function in the interpretation of genetic information, whereas, multi-retention gene function is biased towards response to signals, especially for development and defense. Manual investigation of over 755 enriched GO terms in ‘development’ revealed six and 17 enriched GO terms over-represented in Index 1 and 2 representatives of single and multi-retention genes, respectively. Various reproductive structures (floral organs, seeds) were preferentially observed in Index 2, while embryo and embryo sac ‘development’ were over-represented in Index 1. ‘Development’ for vegetative tissues (leaf, root) was common in both indexes (Figure 5B). Seven and 38 enriched GO terms were manually detected in Indexes 1 and 2, respectively, using the term ‘response to’ (Figure 5C). The multi-retention gene pairs (Index 2) showed more enrichments with this term, including genes responding to phytohormones, nutrients, and other biological stimuli. Many biotic stress defense genes (bacterium, fungus, and insect) were observed in Indexes 1 and 2; however, molecules originating from bacteria and fungus were biased to Index 1 and to Index 2, respectively.

### Fate of homoeologues in the recent genome

**Figure 6:**
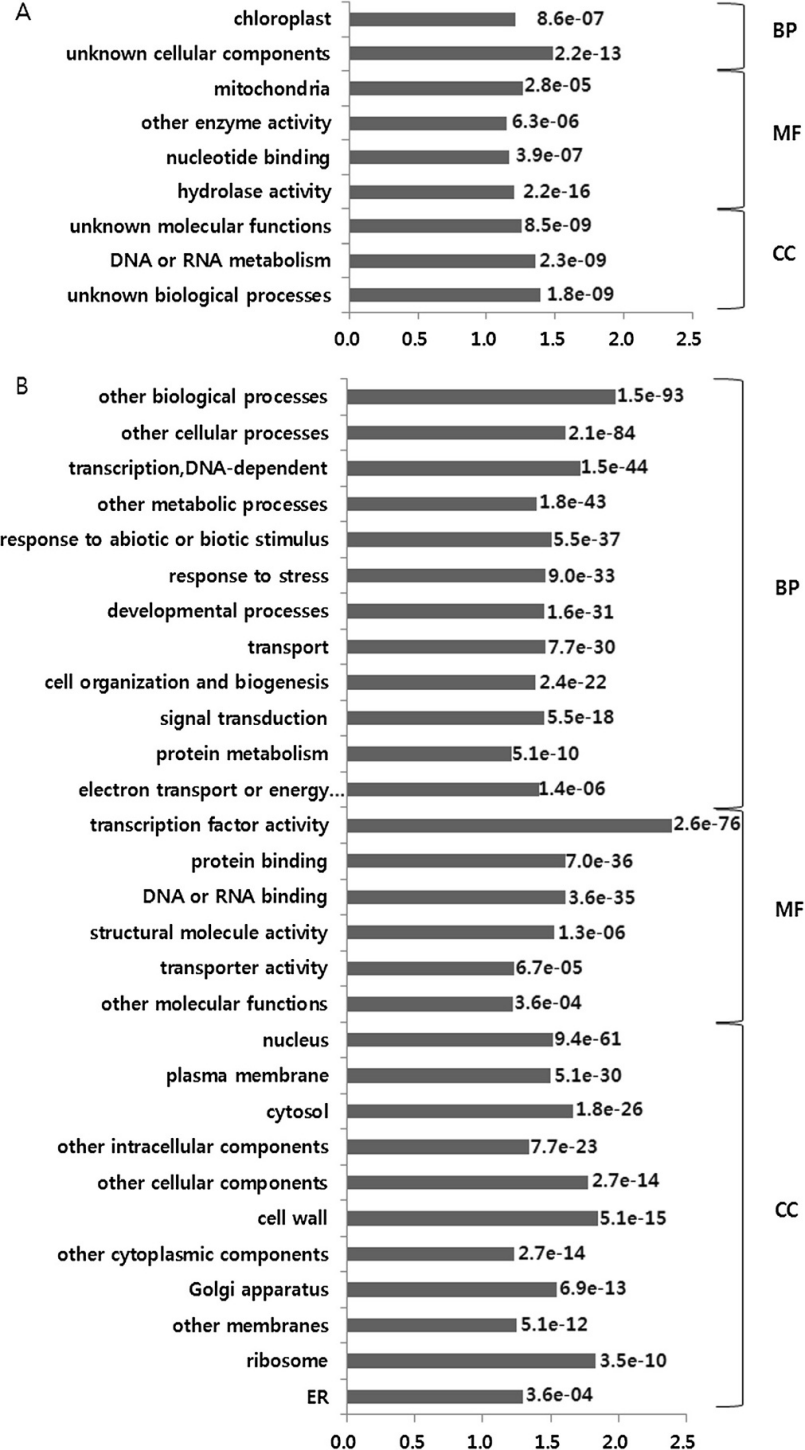
**Enriched GO-slim terms analysed with single and multi-retention homoeologous genes in recent genome.** Overrepresented GO-slim terms for single **(A)** and multi **(B)** retention genes. The x-axis represents X-fold GO-slim enrichment, calculated as the ratio percentages of the cluster frequency of the tested gene set and the cluster frequency of the genomic background. The y-axis represents GO-slim terms. The p-values for fisher’s exact test are indicated on the bar.

The multi-retention genes in the recent genome of the *B. rapa* homoeologues were identified from 18,713 recent genome collinear gene pairs. A total of 10,127 (54.12%) gene pairs were retained as single genes, while 13,302 (35.54%), 5,790 (10.31%), and 20 (0.03%) gene pairs were retained as two, three and four homoeologues, multi-retention genes in the recent genome, respectively. Functional enrichments provided by the single and homoeologous genes were evaluated. The enriched GO-slim terms of single-retention genes in the recent genome were similar to those of recent genome specific genes (Index 1 in Table 3), representing roles in the construction of functional proteins (Figure 6A). The enriched GO-slim terms in multi-retention genes in the recent genome (*B. rapa* homoeologues) were ‘response to stress’, ‘developmental process’ and ‘metabolic process’ (Figure 6B), similar to long-retention genes that had undergone recursive WGD (Figure 5A). These data demonstrate that the major effect of the *B. rapa* WGT event may have been a change in environmental adaptability or in developmental processes. The *B. rapa* homoeologues were analyzed for the existence of different expression patterns and/or positive selection (*K*_*a*_*/K*_*s*_ > 1) (Additional file 5). A total of 145 homoeologues (1.69%) were defined to a “dead” class, showing no expression evidence in mRNA-Seq data. We also defined 690 (8.04%) homoeologues that only had one instance of expression evidence as a “nonfunctionalization” class, and 2,942 (34.27%) homoeologues with spatially differentiated expression patterns as a “subfunctionalization” class (Table 4). The “neofunctionalization” class, containing nine (0.10%) homoeologues was identified with by the existence of both expression evidence and *K*_*a*_*/K*_*s*_ > 1. These results imply that the most frequent evolutionary tract for multi-retention genes following WGT were “subfunctionalization” through a changing in spatial expression patterns (Table 5).

**Table 4:** **Functional differentiation of the** *B. rapa* **homoeologues**

	Retention numbers		
	Two	Tree	Four
Nonfunctionalization	507	183	0
Subfunctionalization	2,026	913	3
Neofunctionalization	8	1	0
Total	2,541	1,097	3

**Table 5:** **Subfunctionalization of** *B. rapa* **genes involved in root and leaf developments**

*B. rapa*	*A. thaliana*	*K*_*a*_/*K*_*s*_	Leaf ^a^	Root ^a^	Stem ^a^	Pool ^a^	Organ ^b^	Absolute expression in*A. thaliana* ^c^	Description
**Root development**									
**Bra033865**	AT1G31930	0.10	1	**8.1e-06**	0.08	1	root	seed, shoot	Extra-large G protein 3
**Bra002904**	AT5G55480	0.21	0.9	**1.1e-04**	8.1e-03	1	root	seed, shoot	Glycerophosphoryl diester phosphodiesterase-like protein (GDPD)
**Bra006834**	AT5G57090	0.07	1	**1.5e-07**	1	1	stamen, root	seed, root	Auxin efflux carrier, root specific role
**Bra030785**	AT1G09560	0.18	1	**1.3e-15**	1	**9.4e-06**	root	root	Germin-like protein 5 (GLP5)
**Bra007924**	AT1G70560	0.10	0.99	**2.9e-04**	0.92	0.83	flower, primary root, root, embryo, leaf, gynoecium, cotyledon,	carpel	Tryptophan aminotransferase of Arabidopsis 1 (TAA1)
**Bra005011**	AT2G39830	0.10	0.99	**6.4e-05**	0.93	1	root, phloem	shoot, petal	DA1-related protein
**Bra007863**	AT2G24260	0.27	1	**4.1e-16**	1	0.96	root hair cell	seed	Basic helix-loop-helix (bHLH)
**Bra005325**	AT2G35610	0.10	0.97	**1.1e-04**	0.21	1	root hair cell	seed, root, silique	Arabinosyltransferase
**Bra002227**	AT5G19320	0.14	1	**1.1e-05**	**1.4e-04**	1	lateral root	root, seed, shoot, flower	RAN GTPase activating protein 2 (RANGAP2)
**Bra008898**	AT5G12330	0.13	1	**5.0e-05**	0.41	1	root	shoot, root	Lateral root primordium 1 (LRP1)
**Bra011079**	AT4G29040	0.02	1	**1.2e-06**	0.01	1	root, root cap, phloem, seedling	sepal, petal, senescent leaf	Regulatory particle AAA-ATPase 2A (RPT2A)
**Bra022183**	AT3G16857	0.09	1	**7.7e-04**	0.43	0.91	primary root, root	seed	Response regulator 1 (RR1)
**Bra030695**	AT1G07630	0.19	1	**1.5e-24**	1	1	lateral root, leaf	sepal, senescent leaf	Protein phosphatase 2C like gene
**Bra014436**	AT3G61440	0.16	0.98	**2.6e-04**	**1.3e-14**	1	root hair cell	cotyledons	Cysteine synthase isomer (CysC1)
**Bra016173**	AT1G70940	0.20	1	**4.2e-06**	0.16	1	root	petal, stamen	PIN3, regulator of auxin efflux
**Bra034623**	AT4G34390	0.26	0.88	**5.6e-04**	0.98	0.89	lateral root	leaf, sepal	Extra-large GTP-binding protein 2 (XLG2)
**Bra019821**	AT1G13260	0.11	0.81	**1.7e-04**	**3.0e-05**	1	flower, lateral root, leaf	root, leaf	EDF4
**Bra022459**	AT3G19820	0.05	1	**3.6e-24**	**1.9e-10**	1	anther, root	shoot, root flower,	Enhanced very-low-fluence responses 1 (EVE1)
**Bra023219**	AT1G31880	0.09	0.98	**2.4e-04**	0.87	1	lateral root, root	shoot, seed	BRX, cell proliferation and elongation in the root
**Bra034413**	AT1G35580	0.04	1	**4.5e-06**	0.97	0.27	root	root	Cytosolic invertase 1
**Bra003506**	AT3G62680	0.35	1	**1.9e-04**	1	1	lateral root	root	Proline-rich protein 3 (PRR3)
**Bra015906**	AT1G74500	0.07	1	**4.6e-05**	1	1	root	root	bHLH, root initiation
**Bra032503**	AT1G04550	0.19	1	**4.5e-10**	0.98	1	root	shoot, carpel, hypocotyl, flower	BDL, auxin-mediated processes
**Bra010735**	AT4G38630	0.05	0.91	**6.6e-04**	**1.0e-04**	1	leaf, post-embryonic root, pollen	seed	Multiubiquitin chain binding protein 1 (MCB1)
**Bra021117**	AT3G15540	0.16	1	**1.1e-04**	0.61	0.96	lateral root, stamen filament	seed, stamen	IAA19, primary auxin-response genes
**Bra029267**	AT5G62340	0.49	**8.2e-14**	**8.4e-06**	1	1	lateral root	root	Plant invertase/pectin methylesterase inhibitor superfamily
**Leaf development**									
**Bra012600**	AT4G18390	0.09	**1.5e-04**	**5.4e-04**	0.95	1	leaf	vegetative rosette	Cycloidea and PCF transcription factor 2 (TCP2)
**Bra027284**	AT3G15030	0.20	**1.4e-05**	1	0.01	1	embryo, leaf	petal	TCP4

^a^Digital expression patterns by Audic’s test, bold characters represent over-expressed tissues with p-value < 0.001. ^b^specific organs, sub-functionalized *B. rapa* genes involved in the developmental process, ^c^expression patterns of A. thaliana in eFP web site (http://bbc.botany.utoronto.ca/efp/cgi-bin/efpWeb.cgi) in developmental map.

## Discussion

### Extraction of three chronological genomic segments from the *B. rapa*genome

The mesohexaploid *B. rapa* genome underwent four rounds of polyploidy events after the diversification of the Eudicots [12]. Three paleo-WGDs, known as At-γ, β, α events (in chronologic order) are shared with the entire core Brassicaceae family, whereas the last genome triplication is specific to the *Brassica* genus [12]. *Brassica* WGT yielded three or six copies of genome colinearity in diploid and amphidiploid *Brassica* species, respectively [2–4, 26]. Advancements in next-generation sequencing (NGS) technology and bioinformatics analyses have increased the number of WGS projects for commercial and/or evolutionarily important plants. However, NGS based approaches often bias genome assembly toward gene rich regions, leaving most intergenic and repetitive regions unassembled [27]. In this study, we used 283.8 Mb of *B. rapa* draft genome (several ‘N’s occur in unassembled regions and unanchored scaffolds), which covered 98% of gene space [5]. This was of sufficient quality to allow our comparative analysis illuminating the effects of WGD/WGT events on *Brassica* gene and genome evolution. There must be some amount of gene loss and underestimation in our present data; however, that should not change the overall evidence leading to our conclusions. As a part of these studies, Cheng et al. (2013) showed that ten chromosomes of the *B. rapa* genome formed from an ancestral tPCK structure (n = 7), revealing one to three copies with GB associations conserved in the ancestral genome, and showing a trace of ancestral centromeres in the *B. rapa* genome [18]. We rebuilt ancestral genomic segments based on the amount of synteny between *A. thaliana* and *B. rapa*, as measured by the average *K*_*s*_ values of each syntenic segment (Figure 2). This was based on the assumption that genes in synteny should share similar substitution rates. These assessments clearly categorize syntenic segments into three chronologic classes: recent, young, and old genomes (Figure 1). The average *K*_*s*_ value of the recent genomic segments (0.53; Table 1) indicate that the birth of the recent genome was concurrent with the split of *A. thaliana* and *B. rapa* (24–40 Mya) [20]. The average *K*_*s*_ value of young (1.16) and old (2.19) genomic segments in *Brassica* were similar to the paralogous gene sets in *Arabidopsis* recent (0.8–1 *K*_*s*_) and old (2.0–2.2 *K*_*s*_) segments [20]. These values suggest that the birth of the young genome in *Brassica* is slightly older than the *Arabidopsis* recent polyploidy event, whereas the old *Brassica* genome is close in birth age to the old *Arabidopsis* polyploidy event (Table 1). Rare traces of the oldest paleo-WGD are explained by the broken collinearity resulting from recursive WGD and fractionation during 120 million years of evolutionary history after the emergence of the Eudicot plants [28].

### Fast evolving genes may mediate stress response or development by changing protein metabolism

Genome-wide screening for fast evolving genes in plants has not been widely pursued. However, several proteins have been reported to belong to rapidly evolving gene families, including the nucleotide binding-leucine rich repeats (NB-LRRs) involved in plant resistance [29], and transcription factors (TFs) [30], as well as several protein-coding genes in plastids involved in RNA polymerase subunits and ribosomal proteins [31]. In this study, a total of 636 potentially fast evolving genes were detected genome-wide. The distribution of the fast evolving genes was different among chronological genomes, chromosomes (Figure 3), and GBs (Additional file 3), suggesting a biased location of these genes. The fast evolving genes identified in our study had a high frequency of multi-retention suggesting that fast evolving genes may be affected by dosage-sensitive genes during recursive WGD events [21]. Our GO terms and enrichment analysis of *B. rapa* fast evolving genes suggest that *B. rapa* has undergone positive evolution through rapid base substitution in these genes, enhancing environmental stress adaptability. The NB-LRR homologues in the whole-genome triplicated *Brassica* ancestor were deleted or lost quickly, and seem to have experienced species-specific amplification by tandem duplication [32, 33]. In our study, only three copies of NB-LRR genes were identified as fast evolving genes (Additional file 3) because tandem duplicated genes were filtered out based on our syntenic analysis criteria. Defense and developmental processes usually have three steps: recognition of signaling (biotic and abiotic stimulus for stress or hormones for development), signal transduction, and the expression of target genes. In our study, the GO-slim term ‘protein modification’ was enriched in fast evolving genes, and contained many sub-terms including the regulation of Ser/Thr signaling, effects on translation, and post-translational modifications enabling actual protein function (Figure 4B). These terms imply that defense and developmental processes may be enhanced in signaling levels and/or functional protein levels. Development and defense system signaling levels have been reported to be tightly linked [34]. The results of our study suggest that stress response and developmental processes may have been enhanced by rapidly changing protein metabolism during the course of *B. rapa* evolution.

### Evolutionary innovations of the recent *B. rapa*genome compared to its ancestral genome

We classified *B. rapa* genes into eight retention patterns (Table 3). The retention rates of *B. rapa* genes were similar to that of *A. thaliana* paralogs [20]. The multiple collinear gene pairs in the recent and young genomes (Index 2) were older than genes specific to the recent genome with a higher standard deviation of *K*_*s*_ values, although both gene sets were classified into the same category of recent genome. Based on this evidence, we estimated functional bias among seven patterns of gene sets in a step-wise manner, excluding genes with lost synteny (Index 5 in Table 3). The results of these analyses suggest that genes retained in specific chronological genomes (Indexes 1 and 6) were enriched with the function of genetic material interpretations, such as DNA/RNA/protein metabolism and transcription (Figure 5A). This functional bias was similar to the single retention genes of *A. thaliana* [24, 35]. However, genes with multiple synteny in different chronological genomes (Indexes 2–4) had frequent GO enrichments in ‘signal transducer’ or ‘transport’ mainly related to defense or development (Figure 5A), implying a more detailed process of adaptive evolution following WGD. Manual inspection of enriched GO terms mainly detected ‘development’ (Figure 5B) or ‘response to stimulus’ (Figure 5C) from the recent genome specific gene set. This data represented the functional innovative patterns following WGD or WGT events. Our study showed that the young genome was enriched with the GO-terms ‘reproductive structures (floral organs, seeds)’ , implying that reproductive organ development may be functionally diversified in the young genome [36]. Interestingly, we observed that the GO terms ‘embryo’ and ‘embryo sac development’ were over-represented in genes specific to the recent genome, while ‘developments for vegetative tissues (leaf, root)’ were shared in two chronological genomes (Index 1 and 2). Embryos contain primordial tissue layers and drive morphogenetic diversity by regulating cell specification and cell-cell communication [37]. Therefore, our GO enrichments cautiously suggest that the morphological diversity of *B. rapa* may be expanded during embryogenesis by concerted evolution. The specific GO enrichment patterns of signal response genes indicate that many pathogen (bacteria, fungi, and insects) or environmental stress (cold, heat, freezing, water deprivation) response genes were over-represented in both categories of Index 1 and 2 (Figure 5C), with duplicates continuously retained during recursive WGD events. Many phytohormones were also enriched in Index 2, which are important in regulating plant developments, as well as in defense by way of cross-talk signal transductions [34, 37, 38]. These retention patterns suggest that the *B. rapa* genome was more innovatively evolved to adapt to biotic/abiotic stress than to phytohormone stimuli.

### Subfunctionalization is the primary fate of multi-retention genes in the recent genome

Functional diversification of surviving genes has been reported to be a major characteristic of long-term evolution in polyploids [24]. Two times as many multi-retention genes were present in the *B. rapa* recent genome than there were in the *A. thaliana* recent genome, after the α-WGD event [24], in our study. There were 3.4 times as many two-copy retention genes than that of three copy retention genes. These results support the two-step theory of WGD events for the *B. rapa* mesohexaploid genome [39]. GO functional annotation enrichments for single- and multi-retention gene sets were biased toward genetic control and the regulation of stress and/or development, respectively (Figure 6). In previous research duplicated genes were reported to acquire new functions via neofunctionalization or to alter their functions via subfunctionalization and pseudogenization [40]. mRNA-Seq data have been published [39] providing tissue and developmental stage specific expression data, which enable us to study subfunctionalization for several developmental stages. We suggest that subfunctionalization is the major drive for the evolution of multi-retention genes (Table 4), because 34.27% of the homologues studied have spatially differentiated expression patterns, In previous research, 50% and 36–49% of homologous genes had undergone subfunctionalization in *Glycine max* and *Triticum aestivum* L., respectively [41, 42]. Comparatively lower distributions of *B. rapa* subfunctionalization were observed because of the limited number of the mRNA-Seq libraries used in this study. *A. thaliana* and *B. rapa* homoeologous gene expression patterns did not completely coincide in spite of their syntenic orthologous relationship (Table 5). Differences in expression patterns suggest a gain or alteration of function in duplicated *B. rapa* genes. Those genes, and/or their homologues, may have acquired new functions or altered their ancestral function after the *Brassica* WGT event. Several genes that we categorized as “dead” or “nonfunctionalization” classes could be expressed in other tissues or under specific conditions because of the dearth of public mRNA-seq data [39]. Tissue-specific genes could be co-expressed in other tissues when expression conditions change. Our study shows a frequent subfunctionalization fate in duplicated genes, with small exceptions of nonfunctionalization via reciprocal gene loss after *B. rapa* WGD events. However, the mechanisms for gene loss, in both subfunctionalization and neofunctionalization, have not been fully resolved. Different duplicate retained gene expression levels were recently reported to be partially a result of epigenetic modifications such as methylation, histone modification, small RNAs, and transposable element genes (review in [43]). The patterns and processes driving gene retention and evolution in *Brassica* will be further elucidated through gene expression and function analysis, combined with epigenetic studies of the paralogous homologues.

## Conclusions

Our interpretation of the *B. rapa* genome, based on sequence diversities, led to our construction of three chronological genomes. Furthermore, we identified fast evolving genes, and single- and multi-retention genes in the recent genome; long retention genes in young/old genomes; and three chronological genome specific genes. Both the fast evolving and the multi-retention genes were enriched with the GO terms ‘stress response’ and ‘development.’ However, detailed functions appeared to be related to the regulation of signal cascades and/or transport systems, rather than in recognition; while gene functions under ‘transcription and translation’ were highly enriched in recent or young genome specific gene sets. High numbers of multi-retention genes in the recent genome had undergone subfunctionalization, rather than neofunctionalization. The results of the present study will be useful in understanding innovative features of the *B. rapa* genome following *Brassica* WGT, and contribute to experimental design for studying *Brassica* diversity.

## Methods

### Construction of syntenic regions

We downloaded the model Brassicaceae plant *A. thaliana*’s genomics resource from The Arabidopsis Information Resource (TAIR, ver. 10) website (ftp://ftp.arabidopsis.org/Sequences/whole_chromosomes/), and the *B. rapa* genome from the BRAssica Database (BRAD, ftp://brassicadb.org/Bra_Chromosome_V1.2/). We identified all possible homologous proteins between *A. thaliana* and *B. rapa* using BLASTp, with a cut-off Expectation Value less than 1e-5, to build syntenic regions. After removing redundancies, the collinear gene-pairs within an adjacent 10 Kb were identified as syntenic segments using MCScan (ver. 0.8). MCScan is able to search for collinear protein pairs throughout a genome [11], and is downloaded from the Plant Genome Duplication Database (PGDD, http://chibba.agtec.uga.edu/duplication/mcscan/). The “mcl” algorithm in MCScan was used with the default parameters “--abc --abc-neg-log -abc-tf ‘mul (0.4343), ceil (200)’” to define syntenic segments.

### Estimation of the levels of *K*_*s*_and *K*_*a*_

Protein sequences for each collinear protein pairs were aligned by clustalw2 and *K*_*s*_ and *K*_*a*_ were estimated using the maximum likelihood method in the PAML and PAL2NAL package [44, 45]. Finally, we applied the Yang-Nielson method.

### Identification of fast evolving genes

The standard deviations of *K*_*s*_ for a syntenic block were calculated using in-house scripts with the equation below


where *x*_*i*_ is *K*_*s*_ for individual gene pairs in a syntenic block and  is average K_s_ for a syntenic block. Z-score and its p-value were calculated with the scipy.stats module in the python package. We defined fast-evolving genes with a p-value less than 0.001.

### GO enrichment analysis

GO terms for *A. thaliana* proteins were downloaded from TAIR web-site (ftp://ftp.arabidopsis.org/home/tair/Ontologies/Gene_Ontology/). GO and GO-Slim terms for *B. rapa* genes were assigned based on their *A. thaliana* syntenic counterpart. GO enrichments were analyzed by Fisher’s exact test (in the python module (ver.0.1.4)) specifying a p-value < 0.001 [46].

### Identifying of *B. rapa*homoeologues and the fate of genes

Homologues were defined as those collinear gene pairs, as filtered by BLASTp and MCScan above, observed in both the *B. rapa* and *A. thaliana* genome. The fate of the *B. rapa* homologues was determined using gene expression pattern and sequence diversification rates compared to pivotal genes. To analyze gene expression patterns the mRNA-Seq data of *B. rapa* was downloaded from the BRAD database (http://brassicadb.org/brad/genomeDominanceData.php), including reads per kilobase of exon model per million mapped reads (RPKM) values as expression evidence from three tissues (leaf, root, and stem of *B. rapa* accession Chiifu-401-42), as well as two pooled mRNA libraries for *B. rapa* Chiifu-401-42, and a cultivar line L 58 [39]. The genes with an RPKM value of “0” were defined as pseudo-genes without expression evidence. Based on RPKM, differentially expressed genes in specific libraries were analyzed using Audic’s test (p-value < 0.001) [47]. Enrichment values were applied to define “subfunctionalization”. “Neofunctionalization” was defined as genes with *K*_*a*_/*K*_*s*_ values larger than one.

## Electronic supplementary material

Additional file 1: **Sequence divergence of collinear gene pairs between** *A. thaliana* **and** *B. rapa* **according to periodic genome segments.** (XLSX 3 MB)

Additional file 2: **Statistics of 24 genomic blocks in** *B. rapa* **genome.** (XLSX 17 KB)

Additional file 3: **List of genes, genomic blocks, statistics, and annotation of the fast evolving genes.** (XLSX 172 KB)

Additional file 4: **GO enrichment according to retention patterns of collinear gene pairs.** (XLSX 77 KB)

Additional file 5: **Subfunctionalization and neofunctionalization patterns of** *B. rapa* **homoeologues.** (XLSX 2 MB)
